# Ratiometric imaging of extracellular pH in *Streptococcus mutans* biofilms exposed to different flow velocities and saliva film thicknesses

**DOI:** 10.1080/20002297.2021.1949427

**Published:** 2021-07-19

**Authors:** Mathilde Frost Kristensen, Ellen Frandsen Lau, Sebastian Schlafer

**Affiliations:** a Department of Dentistry and oral health, Aarhus University, Aarhus, Denmark; b Section for Periodontology, Department of Dentistry and Oral Health, Aarhus University, Aarhus, Denmark

**Keywords:** *Streptococcus mutans*, dental biofilm, pH ratiometry, saliva film, microfluidics

## Abstract

**Introduction:** Fluid flow has a prominent influence on the metabolism of surface-attached biofilms. Dental biofilms are covered by a thin saliva film that flows at different rates in different locations under stimulated and unstimulated conditions.

**Methods:**The present study employed pH ratiometry to study the impact of different flow velocities, saliva film thicknesses and saliva concentrations on microscale pH developments in S*treptococcus mutans* biofilms of different age.

**Results:**While saliva flow at a velocity of 0.8 mm/min (unstimulated flow) had little impact on biofilm pH, stimulated flow (8 mm/min; 80 mm/min) affected vertical pH gradients in the biofilms and raised the average pH in 48-h biofilms, but not in 72-h and 168-h biofilms. The saliva film thickness had a strong impact on biofilm pH under both static and dynamic conditions. pH drops were significantly higher in biofilms exposed to a thin saliva film (≤ 50 µm) than a thick saliva film (> 50 µm). pH drops in the biofilms were also strongly dependent on the saliva concentration and thus the buffer capacity of the salivary medium. For 48-h and 72-h biofilms, but not for 168-h biofilms, pH drops in distinct microenvironments were more pronounced when the local biofilm thickness was high.

## Introduction

Recent advances in fluorescence microscopy have opened new possibilities for the quantification of diffusing molecules in biofilms [1]. With appropriate fluorescent indicators, the concentration of small solutes with a dramatic impact on biofilm metabolism and virulence, such as O_2_ or H^+^ can be visualized in real-time, while preserving the three-dimensional structure and functional integrity of the biofilm [2–5]. Microscopy-based recordings of pH in dental biofilms have demonstrated the presence of steep chemical gradients in the micrometer range and thus challenged traditional concepts of biofilm behavior, which relied on electrode measurements of bulk pH [6].

In a laboratory model of dental biofilm, Xiao et al. could show that three-species biofilms kept at acidic pH were not neutralized homogenously by phosphate buffer [2]. In some bacterial colonies, and in particular in the core of large mushroom-shaped clusters, pH remained low for prolonged periods of time [7,8]. The resistance to neutralization could, at least in part, be attributed to the biofilm matrix, as demonstrated by glucosyltransferase B knock-out mutants and by enzymatic digestion of extracellular polysaccharides (EPS). Schlafer et al. demonstrated, in a five-species model of dental biofilm, that pH microenvironments are not only created by local differences in diffusion properties, but also directly by bacterial acid metabolism [3]. Acid production upon exposure to glucose differed largely in separate areas of the same biofilm, which resulted in distinct niches with pH differences of up to two units. These so-called acidogenic hot spots could later also be identified in *in situ*-grown biofilms [4,9] and seem to be a characteristic of dental biofilms that is closely correlated with disease progression. Recently, Kim et al. were able to show elegantly in a dual-species model that the presence of acidogenic hot spots correlates with increased local sub-surface demineralization of the underlying enamel [10].

While these results shed new light on the caries process and may potentially contribute to identify new targets for disease control, it is important to note that all of the above-cited studies were conducted under static conditions, and hence without applying the constant flow of saliva that supragingival biofilms in the oral cavity are exposed to [11]. The saliva velocity in the mouth varies depending on the location and was estimated to range from 0.8 mm/min for the upper-anterior buccal surfaces, during resting conditions, to 350 mm/min for the lower-anterior lingual surfaces, during stimulated conditions [12,13].

Several classical electrode-based studies have highlighted the importance of saliva flow for biofilm pH. Maxillary sites exhibit a significantly lower pH under resting conditions than mandibular sites [14–16], which may be explained by the estimated 10-fold higher saliva velocity in the mandibula [16]. Following a sucrose challenge, biofilm pH is restored at a speed that depends on the amount and velocity of saliva present [14,17], and masticatory activity increases the saliva flow to an extent that may prevent the pH from dropping below critical values. While sucrose, administrated as a rinse, typically leads to rapid pH drops below 5.5 [18], provision of sucrose in conjunction with a meal or in a chewing gum was shown to result in smaller pH drops [18–20].

These studies clearly illustrate the importance of saliva flow for the maintenance of oral homeostasis [21], and recent microscopy-based experiments demonstrate a crucial impact of flow not only on bulk pH, but also on the microscale pH landscapes inside biofilms. In a pilot study, conducted on a five-species model of dental biofilm, a slow medium flow of 1 mm/min was shown to reverse vertical trans-biofilm pH gradients, rendering the biofilm base more acidic [22]. Moreover, acidogenic microenvironments were conserved better under flow in mature biofilms (120 h) than in younger biofilms (30 h).

In contrast to saliva flow, the effect of the saliva film thickness on biofilm pH has received little attention. Depending on the method employed, the film thickness has been estimated to vary between 10 and 100 µm [23–25]. As the film thickness is proportional to the volume of buffering salivary medium that exchanges protons with the biofilm liquid, it may be an important and hitherto overlooked parameter influencing biofilm pH. We have recently developed a microfluidic flow cell with an adjustable geometry that allows for microscopy-based monitoring of pH inside dental biofilms [26]. The aim of the present work was to investigate the impact of different flow velocities and of saliva film thickness on pH microenvironments in highly standardized *S. mutans* biofilms of different ages.

## Materials and methods

### Biofilm growth

*Streptococcus mutans* (DMS20523) cells were grown in brain-heart infusion broth (BHI) for 18 h at 37°C until late exponential phase. The cultures were then centrifuged (1,150 g, 5 min) and adjusted to an optical density of 0.5 (550 nm) in 0.9% NaCl (pH 7). Equal amounts of bacterial culture and BHI including 5% sucrose were added to petri dishes containing sterilized glass slabs (size: 4 × 4 x 1.5 mm; surface roughness: 1,200 grit; Menzel, Braunschweig, Germany). The petri dishes were incubated for 24, 48, 72 or 168 h at 37°C under aerobic conditions for biofilm development. The medium was replaced on a daily basis with fresh BHI containing 5% sucrose.

### Calibration

Extracellular pH in the biofilms was determined by confocal microscopy with the ratiometric dye SNARF^TM^-4 F 5-(and-6)-Carboxylic Acid (C-SNARF-4; Thermo Fisher^TM^ Scientific, Waltham, MA) [3]. For calibration of the dye, 2-(N-morpholino)ethanesulfonic acid (MES) buffer solutions (50 mM; Sigma-Aldrich, Brøndby, Denmark) were titrated to pH 4–8 in steps of 0.2 pH units, and mixed with C-SNARF-4 (20 μM) in 96-well µ-plates (Ibidi GmbH, Gräfelfing, Germany). An inverted confocal microscope (Zeiss 510 META; Zeiss, Jena, Germany) with a 63 x (1.4 NA) oil immersion objective (Plan Apochromat; Zeiss) was used for image acquisition. C-SNARF-4 was excited with a 543 nm laser line, and emission was detected from 576–608 nm (green channel) and from 629–661 nm (red channel) simultaneously (META detector). The microscope was set to a pinhole size of 2 Airy Units (1.6 µm optical slice thickness), an image size of 364 × 364 pixels (143 x 143 µm^2^), a pixel dwell time of 18.03 µsec, zoom 1 (0.4 µm/pixel) and an 8 bit intensity resolution. For every pH value, images were recorded at 35°C in three fields of view (FOVs) chosen at random. Additionally, an image with the laser turned off was acquired for each pH value and used for background subtraction. All red and green channel images were exported to ImageJ [27,28] as TIFF files, the background was subtracted and the green channel images were divided by the red channel images. The resulting ratios (*R*) were plotted against the respective pH values and fitted to a symmetrical sigmoidal curve (R = 0.998) using an online software (www.mycurvefit.com) (Figure S1):

(1) $$pH={\left[{\left(\frac{2.2815581}{R-0.1293069}\right)}^{\left(\frac{1}{4545673}-1\right)}\right]}^{\left(\frac{1}{8.748894}\right)}\times 34.62357$$

### Ratiometric pH measurements under static conditions

Initially, extracellular pH was monitored in 24-h, 48-h, 72-h and 168-h *S. mutans* biofilms placed in cleared whole saliva under static conditions. Saliva from one healthy volunteer was collected after written informed consent was obtained (Danish National Committee on Health Research Ethics (1–10–72-178-18)). The volunteer had no active caries lesions or periodontal disease, did not use antibiotic or anti-inflammatory medication for three months prior to the study and refrained from eating or drinking for 1 h before collection. Paraffin-stimulated saliva was collected on the day of the experiment, then cleared by centrifugation (1,150 g, 5 min), titrated to pH 7 with 0.2 M HCl and kept on ice. Glass slabs with biofilms were placed in 96-well µ-plates containing cleared stimulated saliva, sucrose (4% w/V) and C-SNARF-4 (20 µM), with the biofilms facing downward. pH images were acquired with the same microscope, settings and temperature as for the calibration. Five FOVs were chosen at random and their x-y-positions marked in the microscope software. Hereafter, images were acquired after 10 and 20 min of exposure to sucrose, 5 µm from the top and at the bottom of the biofilm. Images with the laser turned off were taken at regular intervals for background subtraction.

**Figure 1.**
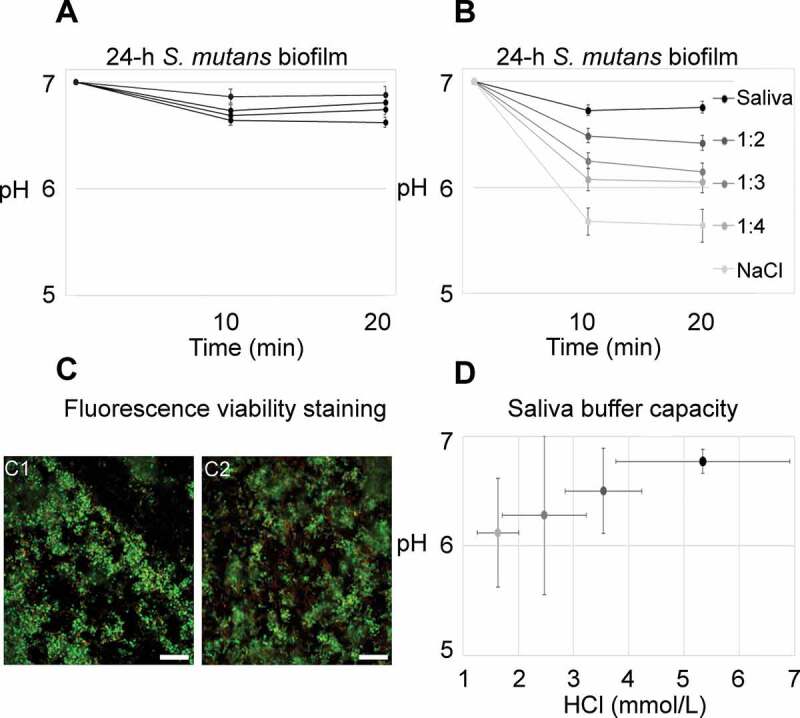
pH developments in 24-h *S. mutans* biofilms under static conditions. **A**) Average pH in 24-h biofilms exposed to whole saliva and sucrose never dropped below 6.5. Each line represents the average pH recorded in the top and bottom layer of five fields of view from one biofilm. Error bars = SD. **B**) Biofilm pH was clearly dependent on the saliva concentration in the medium. Each line represents the average pH of four biofilms, with pH being recorded in the top and bottom layer of five fields of view in each biofilm. Error bars = SD. **C**) The difference in pH could not be attributed to a bactericidal effect of saliva. Viability was high in biofilms kept in whole saliva (67.9% ± 10.2% SD) and in 0.9% NaCl (70.7% ± 12.3% SD), as evidenced by viability staining. Representative images are shown for biofilms kept in saliva (C1) and in 0.9% NaCl (C2). Viable cells appear green, membrane compromised cells red. Bars = 20 µm. **D**) Average pH in the biofilms is positively correlated with the buffer capacity of the surrounding medium. The coding of grey shades is the same as in **B**. Error bars = SD

When incubated in whole saliva, only minor pH drops could be observed in the investigated 24-h biofilms (Figure 1A). Those findings were in contrast to previous experiments that showed consistent pH drops of more than one unit for biofilms incubated with 0.9% NaCl (data not shown). Therefore, additional experiments were conducted to compare the impact of saliva on biofilm pH. 24-h biofilms were placed in whole saliva (pH 7), diluted saliva (1:2, 1:3 and 1:4 in 0.9% NaCl; pH 7) or 0.9% NaCl (pH 7) containing sucrose (4% w/V) and C-SNARF-4 (20 µM), and imaged as described above. All experiments were at least performed in biological quadruplicates. After pH measurements, the biofilms were immediately subjected to viability staining.

### Viability staining

To test whether saliva influenced acid production in the biofilms through a bactericidal effect, the viability of 24-h, 48-h, 72-h and 168-h biofilms was assessed right after pH ratiometry under static conditions. After monitoring the pH for 20 min in cleared saliva, 0.9% NaCl (negative control) or, for 24-h biofilms, diluted saliva (1:2, 1:3 and 1:4), the biofilms were washed five times in 0.9% NaCl and then stained with a live/dead stain (LIVE/DEAD BacLight; Thermo Fisher^TM^ Scientific, Waltham, MA) according to the manufacturer’s instructions. Images were acquired in the central layer of the biofilms in five FOVs chosen at random. A Zeiss LSM 510 META (Jena, Germany) with a 63 x (1.4 NA) oil immersion objective (Plan Apochromat) was used for image acquisition. The dye was excited with 488 nm and 543 nm laser lines, and emission was detected from 500–554 nm (Syto 9, viable cells) and 554–608 nm (Propidium Iodide (PI), membrane-compromised cells). Images were acquired with a pinhole size of 2.28 (syto9) and 2.07 (PI) Airy Units (1.6 µm optical slice), an image size of 364 × 364 pixels (143 x 143 µm^2^), a pixel scan time of 11.17 µsec, zoom 1 (0.4 µm/pixel) and an 8 bit intensity resolution. Experiments were at least performed in biological quadruplicates.

### Buffer capacity testing

Following pH ratiometry of biofilms under static conditions, the buffer capacity of the employed salivary solutions was measured and correlated to the pH drops observed in the biofilms. Each dilution (whole saliva, 1:2, 1:3 and 1:4) was titrated to pH 5.5 with 0.2 M HCl, as measured with a pH electrode (Metrohm AG, Herisau, Switzerland). The buffer capacity (β) was calculated according to equation (2), with *n* being the amount of HCl in mmol/L:

(2) $$\beta =\frac{n}{\Delta pH}$$

### Ratiometric pH measurements under flow conditions

Since 24-h biofilms kept in saliva were not able to lower the pH under static conditions in 96-well plates (Figure 1A), the experiments involving flow were limited to 48-h, 72-h and 168-h biofilms. The experiments performed under static conditions had shown considerable variation in the acidogenic potential of these biofilms. Therefore, acid production inside the biofilms was screened under static conditions in 96-well plates prior to the flow experiments: Biofilms were placed in cleared saliva with sucrose and C-SNARF-4, and the pH in three FOVs was determined ratiometrically after 5 min. Only if a marked pH drop (≥ 0.8 units) was observed, the biofilm was used in the flow experiments.

pH under flow was measured in a custom-made 3D-printed flow-cell. Design and assembly are described elsewhere [26]. Briefly, the flow-cells consisted of a bottomless viewing chamber, an inlet and an outlet. The inlet was connected to a 1 mL syringe (Henke Sass Wolf, Tuttlingen, Germany) containing cleared stimulated saliva, 4% (w/v) sucrose and C-SNARF-4 (20 μM). The outlet was connected to a waste reservoir, and the glass slab with the biofilm was mounted inside the bottomless chamber, with the biofilm facing down. By sealing the bottom of the chamber with a round coverslip (25 mm diameter; Hounisen, Skanderborg, Denmark), a flow-space of 5–120 µm was created between the biofilm surface and the coverslip. The vertical dimension of the flow space was adjusted to either 5–50 µm (low flow space) or 51–120 µm (high flow space) by changing the z-dimension of the printed flow chamber. Laminar flow (Reynolds number = 0.005) was produced by a syringe-pump (TSE Systems 540,060, Bad Homburg, Germany).

In each biofilm, five FOVs were chosen, with a distance of 0.5 mm in between, starting upstream (Figure S2). The exact flow-space for each FOV (the distance between coverslip and biofilm top) was measured with the microscope software. Based on the average flow space (*h*; mm) in the five FOVs and the width of the flow-cell (*b*; 4.2 mm), the desired flow velocities (*v*; mm/min) were converted to volumetric flow rates (*Q*; µL/min) according to equation [3]

(3) Q=v∗b∗h

The biofilms went through different stages of static (S1, S2) and flow (F1, F2, F3) conditions. Initially, biofilms were incubated under static conditions (S1) for 30 min to induce a pH drop. Thereafter, pH was monitored for 30 min at a flow velocity of 0.8 mm/min (F1) to mimic unstimulated flow conditions in the mouth [12]. Then, pH was again monitored under static conditions for 30 min (S2), followed by 30 min at a flowrate of 8 mm/min (F2), which corresponds to stimulated saliva velocities in some areas of the mouth. Images were acquired 5 µm from the top and the bottom of the biofilm in all five FOVs with 15 min intervals (S1-15; S1-30; F1-15; F1-30; S2-15; S2-30; F2-15; F2-30). At the end of the experiment, a flow velocity of 80 mm/min was applied (F3), corresponding to a stimulated, high flow velocity measured in the oral cavity. pH was monitored after 5 min (F3-5) and, if there was enough flow medium left, after 10 min (F3-10).

In the course of an experiment, the pH of the saliva flow medium typically rose, due to CO_2_ evaporation [29]. To estimate the pH rise of the medium in the flow-cell, part of the cleared saliva used in the experiment was kept at 35°C, and its pH was measured at the end of the flow-experiment with a pH electrode (Metrohm AG, Herisau, Switzerland). Experiments were performed in duplicate for each biofilm age and flow space.

### Digital image analysis

#### pH ratiometry

For calculation of extracellular pH, green and red channel images were exported separately as TIFF files to ImageJ. Background values were subtracted, and a mean filter (pixel radius 1) was applied to compensate for detector noise. For one image from each FOV, typically the green channel image taken at S30, the area identified as extracellular matrix was recognized by intensity thresholding, selected and saved as a region of interest (ROI). The ROI was hereafter transferred to all other images of the same FOV. Then all green channel images were divided by the corresponding red channel images. In these ratiometric images, the average fluorescence intensity (*R*) was calculated inside the ROI and then converted to pH-values according to equation (1).

#### Viability

For quantification of bacterial viability, BacLight images were converted to TIFF files and the green (G1) and red channel images (R1) were imported separately into the digital image analysis software daime [30]. Hereafter, stained bacteria were identified in both color channels by image segmentation with intensity thresholding (G2; R2). To eliminate double stained cells, red channel images were subtracted from the corresponding green channel images (G2-R2 = G3), which were then segmented again to determine the area covered by viable bacteria (G4). Red channel images were processed in the same way (R2-G2 = R3) to calculate the area covered by membrane-compromised bacteria (R4). Thereafter, object masks were extracted from R4 and G4 (R5; G5) and corresponding images were added to each other to determine the total area covered by bacteria (R5+ G5 = T5). The fraction of viable bacteria in each image was then calculated by dividing the area in G4 images by the area in T5 images.

### Statistical analyses

Statistical analyses were performed to test the influence of biofilm age, medium buffer capacity, bacterial viability, biofilm thickness, bacterial area coverage, screening pH and flow space on average biofilm pH and pH at the FOV level. Moreover, the influence of biofilm age and saliva concentration on bacterial viability was tested. Data from two independent variables were plotted as scatterplots and the strength of linear relationships was analyzed. Gaussian distribution of the data was tested by the D’Agostino-Pearson omnibus normality tests. For data sampled from Gaussian distribution, parametric Pearson correlation tests were used. If the data did not follow Gaussian distribution, non-parametric Spearman correlations were the analysis of choice. Results with a p-value < .05 were interpreted as significant. Pearson rank or Spearman rank 95% confidence intervals and coefficients of determination (R^2^) are reported next to the p-values. The software GraphPad was used for all analyses (GraphPad Software, San Diego, CA, USA).

## Results

### Biofilm growth

*S. mutans* formed stable biofilms consisting of dense bacterial clusters, interspersed with regions of lower cell density and cell-free areas. The average biofilm thickness increased constantly over time, from 36 µm (±15 SD) after 24 h to 72 µm (±25 SD) after 168 h, as determined by confocal microscopy (Figure S3). Cell viability in the biofilms was stable (50–70%) and did not correlate with biofilm age (*p* = .18 [−.17, .72], R^2^ = .12).

### pH developments under static conditions

#### 24-h S. mutans biofilms

In the presence of whole cleared stimulated saliva with sucrose, only minor pH drops (< 0.5 pH units) were observed inside 24-h biofilms. Average recordings for each biofilm are shown in Figure 1A. Figure S4 shows the pH in individual FOVs of a representative biofilm. Experiments with biofilms kept in diluted saliva or 0.9% NaCl clearly showed that saliva’s effect on biofilm pH was concentration-dependent (Figure 1B; Figure S5). The critical pH of 5.5 was only reached in some FOVs of biofilms kept in 0.9% NaCl (Figure S5). While the saliva concentration was not correlated with bacterial viability in the biofilms (*p* = .22 [−.21, .70], R^2^ =.10; Figure 1C), the average biofilm pH clearly depended on the buffer capacity of the saliva medium (Figure 1D), which indicates a buffering rather than an antibacterial effect of saliva.

#### *48-h, 72-h and 168-h* S. mutans *biofilms.*

**Figure 2.**
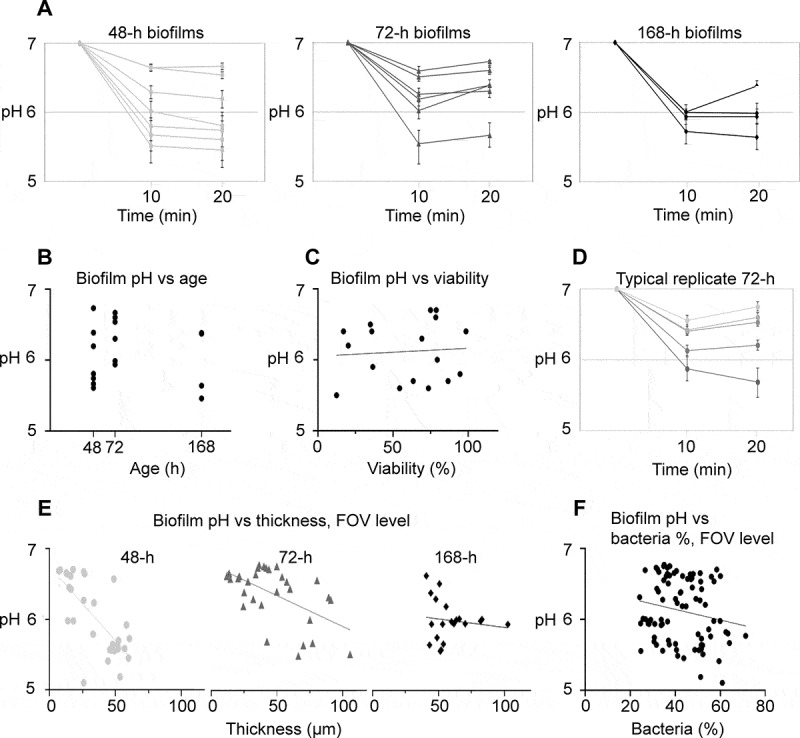
pH developments in 48-h, 72-h and 168-h *S. mutans* biofilms under static conditions. **A**) pH drops in 48-h, 72-h and 168-h biofilms exposed to saliva and sucrose were more pronounced than in 24-h biofilms, but a considerable degree of variation could be observed between individual biofilms. Each line represents the average of pH recordings in the top and bottom layer of five fields of view (FOVs) in a biofilm. Error bars = SD. **B**) pH in the biofilms was not correlated to biofilm age. Each dot represents the average pH in one biofilm. **C**) No correlation was observed between average biofilm pH and bacterial viability, as determined by BacLight staining in five FOVs in each biofilm. **D**) Local biofilm pH varied considerably between different FOVs inside a single biofilm. Each line represents the pH recorded in one FOV in a representative 72-h biofilm. Error bars = SD. **E**) pH in a specific FOV of a biofilm was correlated to local biofilm thickness for 48-h and 72-h biofilms, but not for 168-h biofilms. Each symbol represents the local pH and biofilm thickness in one FOV. **F**) A tendency was observed between local pH in an FOV and the relative bacterial area coverage at the bottom of the biofilm, but the correlation failed to reach the level of statistical significance

Compared to 24-h biofilms, pH drops for 48-h, 72-h and 168-h biofilms in saliva were more pronounced under static conditions (Figure 2A). The pH drops occurred quickly, within 10 min upon exposure to sucrose, after which only minor changes in average pH were observed (10 min: 6.08 vs. 20 min: 6.12). In some biofilms, a slight increase in pH was observed after 10 min, which may be explained by the increase in saliva pH in the course of an experiment due to CO_2_ evaporation. Interestingly, the acidogenic potential was not positively correlated with biofilm age (*p* = .61 [−.58, .37], R^2^ .02; Figure 2B). Average pH (±SD) after 20 min did not differ significantly between 48-h, 72-h and 168-h biofilms (6.08 ± 0.47, 6.35 ± 0.37 and 5.98 ± 0.30, respectively). In contrast, the acidogenic potential varied considerably between individual biofilms of the same age. Average pH after 20 min ranged from 5.46 to 6.65 for 48-h biofilms and from 5.66 to 6.73 and 5.64 to 6.37 for 72-h and 168-h biofilms, respectively. For all biofilms, the average pH inside a biofilm was not correlated with cell viability (*p* = .78 [−.42, .54], R^2^ = .01; Figure 2C). Average pH was positively correlated with average biofilm thickness for 48-h biofilms (48-h: *p* = .0023 [−1, −.68], R^2^ = .92), but not for 72-h and 168-h biofilms (72-h: *p* = .12 [−.96, .25], R^2^ = .49; 168-h: *p* = .59 [−.91, .98], R^2^ = .17).

Although the biofilms consisted of one species only, considerable variation of pH was observed between different FOVs inside the same biofilm, with differences of up to 1.7 pH units. A representative example of a 72-h biofilm is shown in Figure 2D. pH drops in different FOVs of a biofilm were positively correlated with the local biofilm thickness for 48-h and 72-h biofilms, but not for 168-h biofilms (48-h: *p* = < .0001 [−.86, −.49], R^2^ = .52; 72-h: *p* = .0017 [−.76,-.24], R^2^ = .30; 168-h: *p* = .59 [−.54, .33], R^2^ = .02; Figure 2E). An increased bacterial area coverage in the bottom layer of an FOV was associated with lower pH after 10 min, but the correlation did not reach the level of statistical significance (*p* = .09 [−.39, .03], R^2^ = .03; Figure 2F).

### pH under flow conditions

#### *48-, 72- and 168-h* S. mutans *biofilms.*

The experiments performed under static conditions demonstrated that 24-h biofilms were not able to overcome the buffering capacity of whole saliva. The acidogenic potential of older biofilms varied considerably, with other factors than biofilm age, viability and thickness influencing pH developments. Previous experiments conducted under flow conditions with *in situ-*grown biofilms had shown that a saliva flow rate of 5 mm/min neutralized pH in all areas of the biofilms if the pH drops observed under static conditions were moderate [26]. Therefore, experiments under flow were limited to 48-h, 72-h and 168-h biofilms that showed high acidogenicity in a screening step (Figure S6). During pilot experiments with these biofilms under flow-conditions, we observed a potential relationship between the thickness of the flowing saliva film and biofilm pH, i.e. a low flow space correlated with a low pH in the biofilm (Figure S7). Hence, the effect of flow on biofilm pH was investigated in flow cells providing either a low (5–50 µm) or a high (51–120 µm) flow space.

**Figure 3.**
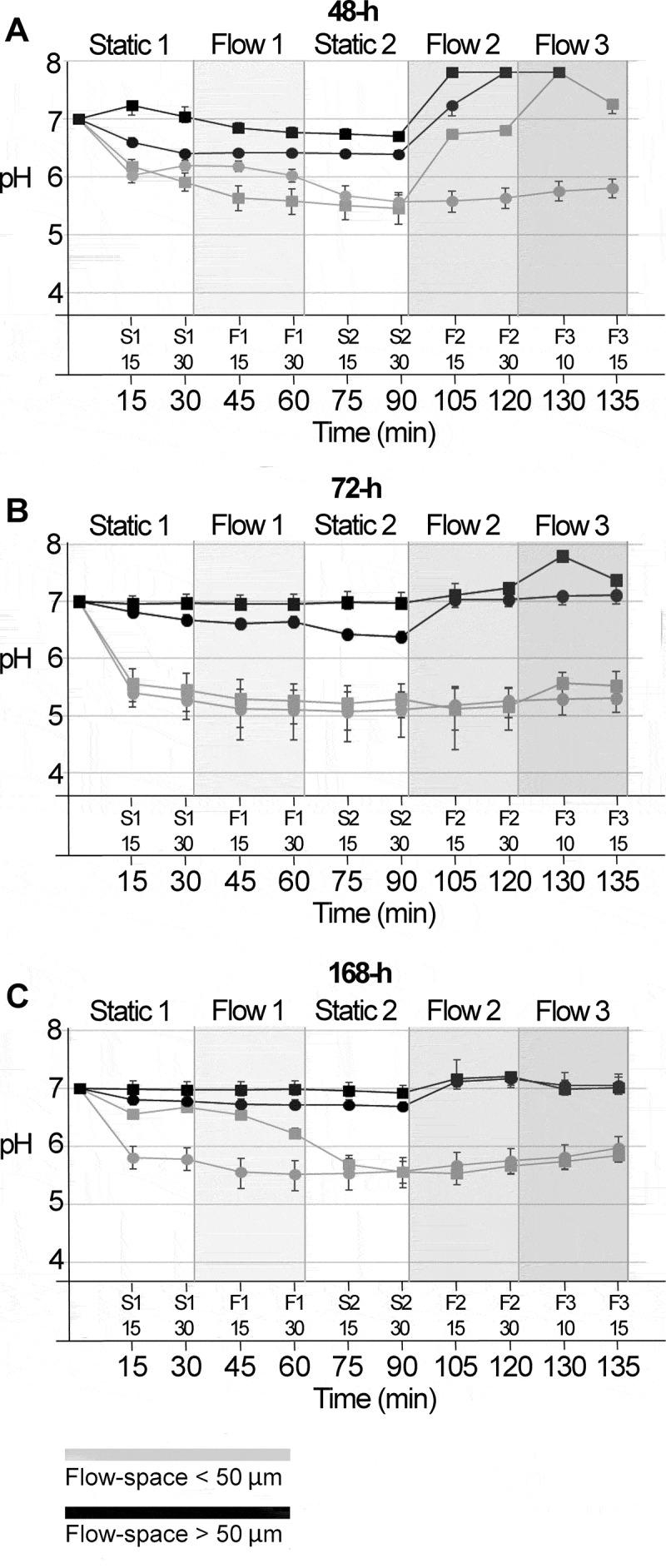
pH developments in 48-h, 72-h and 168-h *S. mutans* biofilms under dynamic conditions. pH was recorded ratiometrically for a total of 135 min after exposure to sucrose in 48-h biofilms (**A**), 72-h biofilms (**B**) and 168-h biofilms (**C**). Initially, measurements were performed under static conditions for 30 min (S1). Thereafter, a flow rate of 0.8 mm/min was applied for 30 min (F1), followed by a second static phase (30 min, S2). Then, a flow rate of 8 mm/min was applied for 30 min (F2), followed by 15 min with a flow rate of 80 mm/min (F3). Each line represents the average of the pH recordings in the top and bottom layer of five fields of view in a biofilm. Grey and black lines show pH developments in flow cells providing a low (≤ 50 µm) or high (> 50 µm) flow space, respectively. Of all parameters investigated, the flow space had the most pronounced effect on biofilm pH. Error bars = SD

In biofilms mounted in flow cells with a high flow space, only minor pH drops were observed, irrespective of biofilm age (Figure 3 A-C, black lines). Average pH in both the initial static phase (S1) and the subsequent flow phases (F1, F2, F3) never dropped below 6.4, for any of the investigated biofilms. The application of a low flow rate of 0.8 mm/min (F1) had little impact on pH levels in these biofilms. Average pH (±SD) values (F1-30) were almost identical to those measured after 30 min of static incubation (S1-30), for all biofilm ages (48-h: S1-30 = 6.7 ± 0.1, F1-30 = 6.6 ± 0.07; 72-h: S1-30 = 6.8 ± 0.1, F1-30 = 6.8 ± 0.1; 168-h: S1-30 = 6.9 ± 0.1, F1-30 = 6.8 ± 0.1). When stimulated flow was applied (F2, F3), pH rose to slightly alkaline values, which is readily explained by the increase in pH of the saliva flow medium in the course of an experiment. The effect of 8 mm/min (F2) or 80 mm/min of flow (F3) on biofilm pH was most pronounced for 48-h biofilms, with a relative increase in average pH (±SD) of 0.95 (±0.05) units from S2-30 to F2-30 (Figure 3A). In comparison, pH only increased by 0.53 (±0.14) and 0.27 (±0.08) units from S2-30 to F2-30 in 72-h and 168-h biofilms, respectively (Figure 3B, C).

Biofilms mounted in flow cells with a low space showed more pronounced pH drops (Figure 3 A-C, grey lines). After 30 min of exposure to sucrose under static conditions (S1-30), average pH (±SD) had reached 6.1 (±0.1), 5.4 (±0.3) and 6.2 (±0.1) for 48-h, 72-h and 168-h biofilms, respectively. During the application of a low flow rate of 0.8 mm/min (F1), average pH dropped further in biofilms of all ages, and the lowest pH levels were reached in the second static phase (S2), with averages of 5.5 (±0.2), 5.2 (±0.3) and 5.6 (±0.2). As observed for biofilms exposed to a high flow space, the application of higher flow rates (F2, F3) on biofilm pH had most pronounced effects on 48-h biofilms (Figure 3A), with a relative increase of 0.7 (±0.5) units from S2-30 to F2-30, compared to changes of −0.02 (±0.01) and 0.1 (±0.1) units for 72-h and 168-h biofilms (Figure 3B, C). All older biofilms preserved low levels of pH, even under the highest flow conditions applied. Confocal microscopy imaging confirmed that the biofilms were not disrupted and did not detach at a flow of 80 mm/min, at least in the examined FOVs.

**Figure 4.**
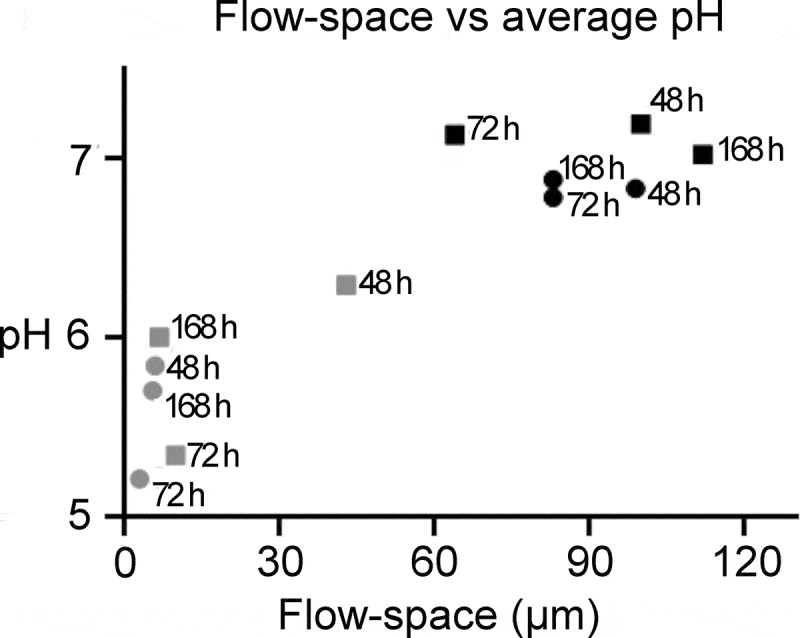
The influence of the flow-space on biofilm pH. A strong correlation was observed between the average pH measured in a biofilm and the flow space. A high flow space and thus saliva film thickness resulted in low pH drops in the biofilms. Grey and black symbols represents the average pH in biofilms exposed to low (≤ 50 µm) and high (> 50 µm) flow-space, respectively

**Figure 5.**
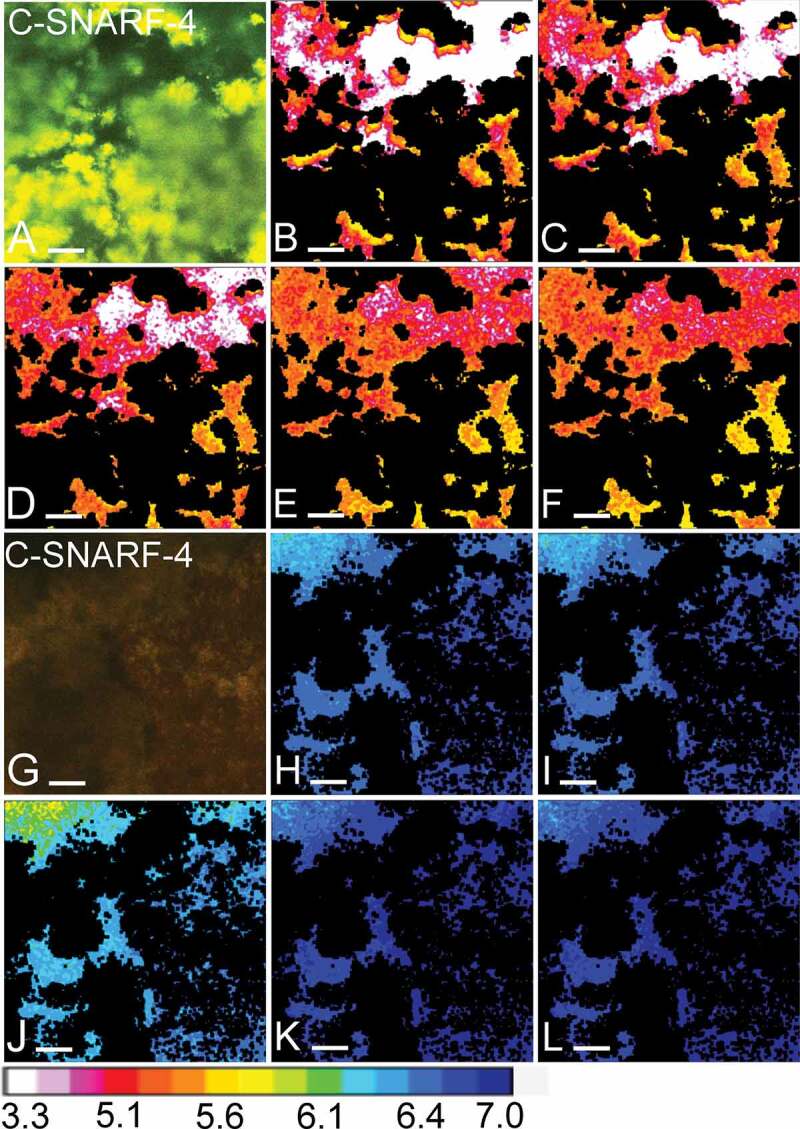
Visual representation of pH developments over time. Panels **A-F** show the pH in one field of view (FOV) of a 72-h biofilm exposed to a low flow space (5 µm), as recorded by pH ratiometry. Panels **G-L** show the pH in an FOV of another 72-h biofilm, exposed to a high flow space (83 µm). **A** and **G** show an overlay of the fluorescence recorded in the green and red channels from the C-SNARF-4 stained biofilms. In the biofilm with a low flow space, the green fluorescence dominates, indicating a low pH, whereas the red fluorescence dominates in the biofilm with a high flow space. In panels **B-F** and **H-L**, bacteria were removed from the images and the pH in the biofilm matrix was visualized using a lookup table (16 colours). After 30 min of exposure to sucrose under static conditions (S1-30), pH dropped to 5.3 ± 0.2 SD in the first FOV (**B**). With the onset of 0.8 mm/min of flow (F1-30), pH dropped to 5.1 ± 0.3 SD (**C**). pH remained unchanged during the second static phase (S2-30: 5.1 ± 0.3 SD, **D**) and increased moderately after exposure to higher flow rates of 8 mm/min (F2-30: 5.3 ± 0.2 SD, **E**) and 80 mm/min (F3-15: 5.3 ± 0.2, **F**). In the second FOV, only minor pH drops were observed in the first static phase and after the onset of 0.8 mm/min of flow (S1-30: 6.7 ± 0.05 SD, **H**; F1-30: 6.6 ± 0.05 SD, **I**). After a moderate pH drop during the second static phase (S2-30: 6.4 ± 0.1 SD, **J**), pH in the FOV was neutralized by the onset of 8 mm/min and 80 mm/min of flow (F2-30: 7.03 ± 0.03 SD, **K**; F3-15: 7.1 ± 0.03 SD, **L**). Bars = 20 µm

Average pH in the examined biofilms was not correlated with biofilm age (*p* = .99 [−.57, .57]; Figure S8A) or the average biofilm thickness (*p* = .62 [−.47, .68]; Figure S8B). Likewise, no correlation was observed between pH recorded during the screening of the biofilms before mounting them in the flow cells, and average pH during the flow cell experiments (*p* = .80, [−.51, .62], R^2^ = .01; Figure S8C). In contrast, pH across all investigated biofilm ages was highly correlated with the size of the flow space (p = < .0001, [.71, .98], R^2^ = .83; Figure 4). Average pH (±SD) in biofilms exposed to a low flow space was considerably lower than pH in biofilms exposed to a high flow space (5.7 ± 0.4 vs. 7.0 ± 0.4). Representative examples of pH developments over time in biofilms with either low or high flow space are shown in Figure 5, as visualized by pH ratiometry.

**Figure 6.**
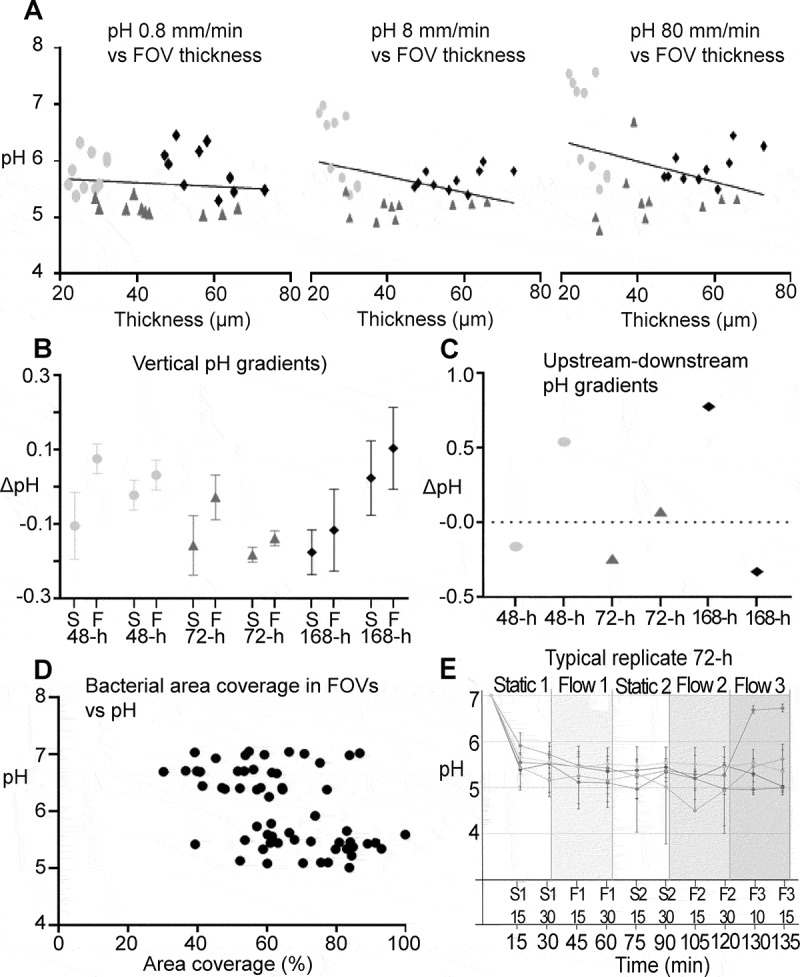
Local pH gradients in 48-h, 72-h and 168-h *S. mutans* biofilms under dynamic conditions. **A**) At flow rates of 0.8 mm/min, 8 mm/min and 80 mm/min, pH in different fields of view of a biofilm was not correlated with the local biofilm thickness. **B**) Under static conditions (S), vertical pH gradients in the biofilms were small, with a tendency towards lower pH in the top layer of the biofilms (negative ΔpH, (S) symbols). With the onset of 8 mm/min of flow (F) pH in the top layer of the biofilms increased compared to pH in the bottom layer (ΔpH of (F) more positive compared to (S)). Each symbol represents the average vertical pH gradient of five FOVs in a biofilm. Error bars = SD. **C**) Horizontal pH gradients between FOVs that were situated upstream or downstream in the flow cells did not follow a systematic pattern. **D**) No correlation could be observed between area coverage at the bottom of a FOV and local pH. **E**) pH recordings from different FOVs of a representative 72-h biofilm illustrate the horizontal pH gradients in the biofilms. pH was recorded under static conditions (S1, S2) and under dynamic conditions with 0.8 mm/min (F1), 8 mm/min (F2) or 80 mm/min of flow (F3). Each line represents one FOV. Error bars = SD

The variation of pH between different FOVs inside the same biofilm was similar to the one observed during experiments in 96-well plates under static conditions. pH differences between individual FOVs were conserved after the onset of 0.8, 8 and even 80 mm/min of flow. Under dynamic conditions, pH in an FOV was not correlated with the local biofilm thickness, for neither 0.8, 8 nor 80 mm/min of flow (Figure 6A). After applying a low flow rate of 0.8 mm/min, vertical gradients did not change significantly (ΔpH = 0.08 ± 0.14 SD). However, with the onset of a flow of 8 mm/min, pH in the top layer of the biofilms increased compared to pH in the bottom layer of the biofilms, which became more acidic in comparison (ΔpH = 0.10 ± 0.09 SD; Figure 6B). No significant difference in pH between FOVs that were situated upstream or downstream (FOV1 vs. FOV5; Figure S3) was observed (Figure 6C). Likewise, no correlation could be observed between the bacterial area coverage in the bottom layer of an FOV and pH (Figure 6D). A representative example of typical pH developments in different FOVs of a 72-h biofilm is shown in Figure 6E.

## Discussion

The present study is the first to investigate the impact of different saliva flow rates and saliva film thicknesses on biofilm pH developments at the microscale. Our results show that pH-values inside *S. mutans* biofilms are influenced by the flow velocity, but even more so by the thickness of the saliva film.

In some regards, the experimental setup of our study mimicked the conditions in the oral cavity very closely. In contrast to other laboratory studies investigating the acidogenic behavior of dental biofilms [2–4,7,9,10,22], we used freshly collected whole cleared stimulated saliva as the experimental medium during incubation. Our experiments performed on 24-h biofilms under static conditions clearly demonstrate that biofilm pH is directly related to the buffering capacity of the employed medium. Average pH values inside the biofilms were strongly correlated with the concentration of saliva around the biofilm, but not with bacterial viability (Figure 1C). This might indicate that pH recordings reported in studies using other incubation media than whole saliva, including some studies from our laboratory, should be interpreted with care. Relative pH differences between individual biofilms, participants, or treatment groups may be valid, but the measured absolute pH values may not reflect the true acidogenic potential of the investigated biofilms [2–4,7–10,22,31–35]. Our data strongly encourage the use of whole saliva for laboratory studies on dental biofilm pH values.

In addition to the incubation medium, our study aimed to reproduce typical flow velocities encountered in the oral cavity under unstimulated and stimulated conditions. As biofilms have a complex geometric structure, calculation of the exact flow velocity in a particular microscopic FOV proved impossible. We used the average of the flow spaces measured in the examined FOVs to determine the applied volumetric flow rate, and the local flow velocity in a particular FOV likely differed to some extent from the mean velocities reported in the manuscript. Moreover, we were unable to change the flow medium during an experiment without risking the formation of air bubbles. Therefore, we used stimulated saliva to monitor pH both at stimulated (8 mm/min or 80 mm/min) and unstimulated (0.8 mm/min) flow rates throughout the study. In most previous investigations that employed microscopy-based methods for biofilm pH recordings, experiments were performed under static conditions [2–4,7–10,31,32,34]. In the past decade, research has become increasingly attentive to the influence of liquid flow not only on biofilm growth, morphology and detachment [36], but also on the distribution of small solutes inside biofilms [37]. Regulatory processes, such as quorum sensing, have been shown to be influenced by both horizontal and vertical gradients of autoinducers that were created by flow [38,39].

In a previous study showing proof-of-principle data on the impact of flow on pH in 96-h *in situ*-grown biofilms from a single subject, the application of 5 mm/min of flow neutralized pH in all areas of the biofilms [26], despite a continuous supply of sucrose. Similarly, a low flow rate of 1 mm/min, applied in a five-species model of dental biofilm, raised the pH in young (30-h) biofilms considerably (from 6.06 to 6.70). In contrast, pH in older (120-h) biofilms continued to drop at the same flow rate (from 6.68 to 6.25) [22]. In the present study, the average pH-values in 48-h, 72-h and 168-h biofilms continued to drop at a salivary flow rate of 0.8 mm/min, whereas higher flow rates (8 mm/min or 80 mm/min) led to a certain increase of average pH values in 48-h biofilms, but less so in 72-h and 168-h biofilms (Figure 3). Differences in biofilm composition and acidogenicity, as well as different geometries of the employed flow cells and differences in the applied flow rates and media render a direct comparison of these results from different studies difficult. However, there seems to be a trend that older biofilms, although not necessarily more acidic under static conditions, are better able to preserve low pH microenvironments during increasing flow.

In the present study, we observed slightly higher pH drops in the top layer than in the bottom layer of the biofilms under static conditions, a trend that was also found in the five-species model [22]. Likewise, Hwang et al. observed more pronounced pH changes in the outer layers of *S. mutans* biofilms that were exposed to neutralizing buffer, and afterwards to sucrose [7]. While the lowest flow rate of 0.8 mm/min had little impact on vertical gradients in the present work, the application of 8 mm/min of saliva flow had a bigger influence on biofilm pH in the top layer than in the bottom layer, which became, relatively, more acidic (Figure 6B). In a previous study that investigated pH in dental biofilms using microelectrodes, similar but far more pronounced vertical gradients were detected under flow, albeit in thick *in situ*-grown biofilms with a height of several 100 µm [40]. Overall, these findings indicate that the surface layers of dental biofilms are more susceptible to metabolic changes induced by diffusive and convective processes.

Previous work has shown that biofilm metabolites, i.e. autoinducers, accumulate downstream under flow [38,39]. We investigated whether the proton concentration increased along the stream in *S. mutans* biofilms by comparing upstream and downstream pH in the flow cells, but did not observe a systematic pattern (Figure 6C). The flow velocities employed in our study may be too low to provoke such horizontal gradients, or else the pronounced pH differences between distinct microenvironments in the biofilms may override any gradients that occur by downstream accumulation of acids.

Although mono-species biofilm models have a number of limitations, such as the lack of inter-species interactions and a reduced complexity of the biofilm matrix, we chose to grow *S. mutans* biofilms for the present study, primarily for reasons of reproducibility. Previous research has demonstrated a high degree of variability in the amount of biofilm formed *in situ* [41], and also in the acidogenic potential of biofilms from different participants under static conditions [9]. To properly study the impact of saliva flow and film thickness on biofilm pH, we used *S. mutans*, which is an avid biofilm former with considerable matrix production and a high degree of acidogenicity. We employed highly standardized growth conditions, which resulted in consistent biofilm viability and thickness, as well as in a continuous increase of the biovolume with growing age (Figure S3). Interestingly, the acidogenic potential of these highly standardized biofilms varied considerably, even under static conditions (Figure 2A). There was no relationship between average pH and biofilm age or bacterial viability (Figure 2B, C), and a positive correlation between the measured pH drops and biofilm thickness was only observed for 48-h biofilms. Future research may explore the influence of additional parameters on biofilm pH, such as bacterial vitality and the composition and structure of the biofilm matrix.

pH microscopy offers the unique advantage of providing pH recordings in different locations of a single biofilm. In accordance with a previous study [7], we observed a high degree of variability in pH between different FOVs inside a biofilm, despite the use of monospecies biofilms (Figure 2D). Analyses at the FOV-level identified a positive correlation between local pH drops and biofilm thickness for 48-h biofilms, but also for 72-h biofilms. In 168-h biofilms, pH seemed to be independent of the local biofilm thickness (Figure 2E), which may indicate that other factors, such as the metabolic state of the bacteria or the three-dimensional structure of the biofilm matrix become more important for pH with growing biofilm age. Moreover, we observed a tendency towards lower pH in FOVs with a high bacterial density in the bottom layer of the biofilm, but the correlation failed to reach the level of statistical significance (Figure 2F). Further work is required to explore the complex interplay between biofilm architecture at the microscale and virulence.

An important observation in the present study was that the space between the biofilm top and the cover glass of the flow cell had a strong impact on biofilm pH (Figure 4). The saliva film thickness in the oral cavity has been determined experimentally to vary between 10 µm and 100 µm [23–25] but to our knowledge, its effect on biofilm pH has not previously been tested in other studies. The marked differences observed between pH in biofilms exposed to either low or high flow spaces could not be attributed to other factors, such as the biofilm thickness or the acidogenic potential of the biofilms, which was determined in a screening step (Figure S6). It seems that the liquid column itself and thereby the volume of saliva that interacts with the biofilm plays a decisive role for pH developments inside the biofilms under both static and dynamic conditions. The results of the present study derive from monospecies biofilms and need to be tested *in situ*, but they indicate that the saliva film thickness may be a hitherto overlooked factor of importance for biofilm pH and, potentially, the development of caries lesions.

## Supplementary Material

Supplemental Material
