# Supplementary figures and images for: Ratiometric imaging of extracellular pH in Streptococcus mutans biofilms exposed to different flow velocities and saliva film thicknesses
Source: J Oral Microbiol. 2021 Jul 19;13(1):1949427. doi: 10.1080/20002297.2021.1949427 (PMC8291056; doi:10.1080/20002297.2021.1949427)

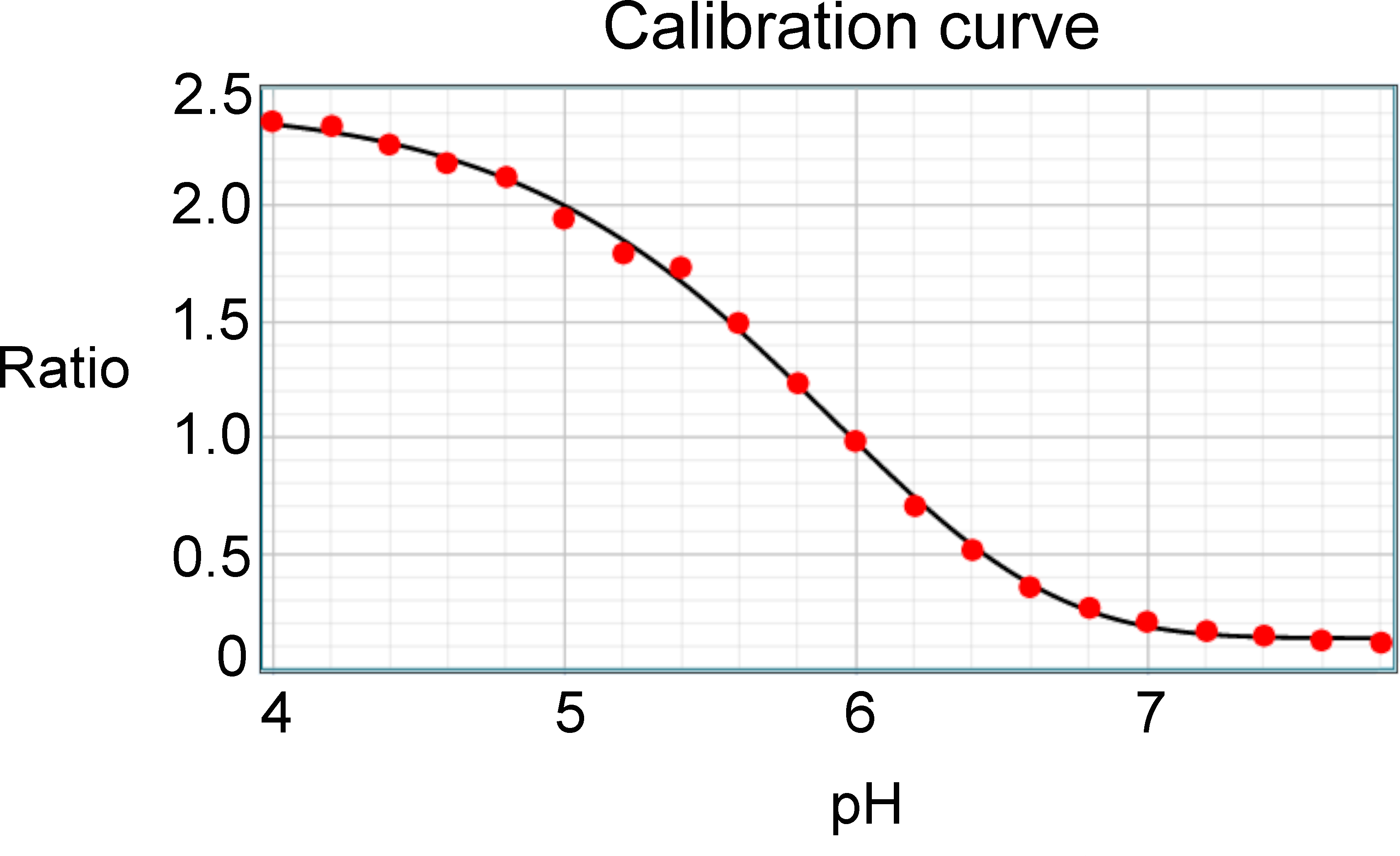

Supplement: Supplemental Material [file ZJOM_A_1949427_SM8111.zip › Supplementary/FIGS1.tif]

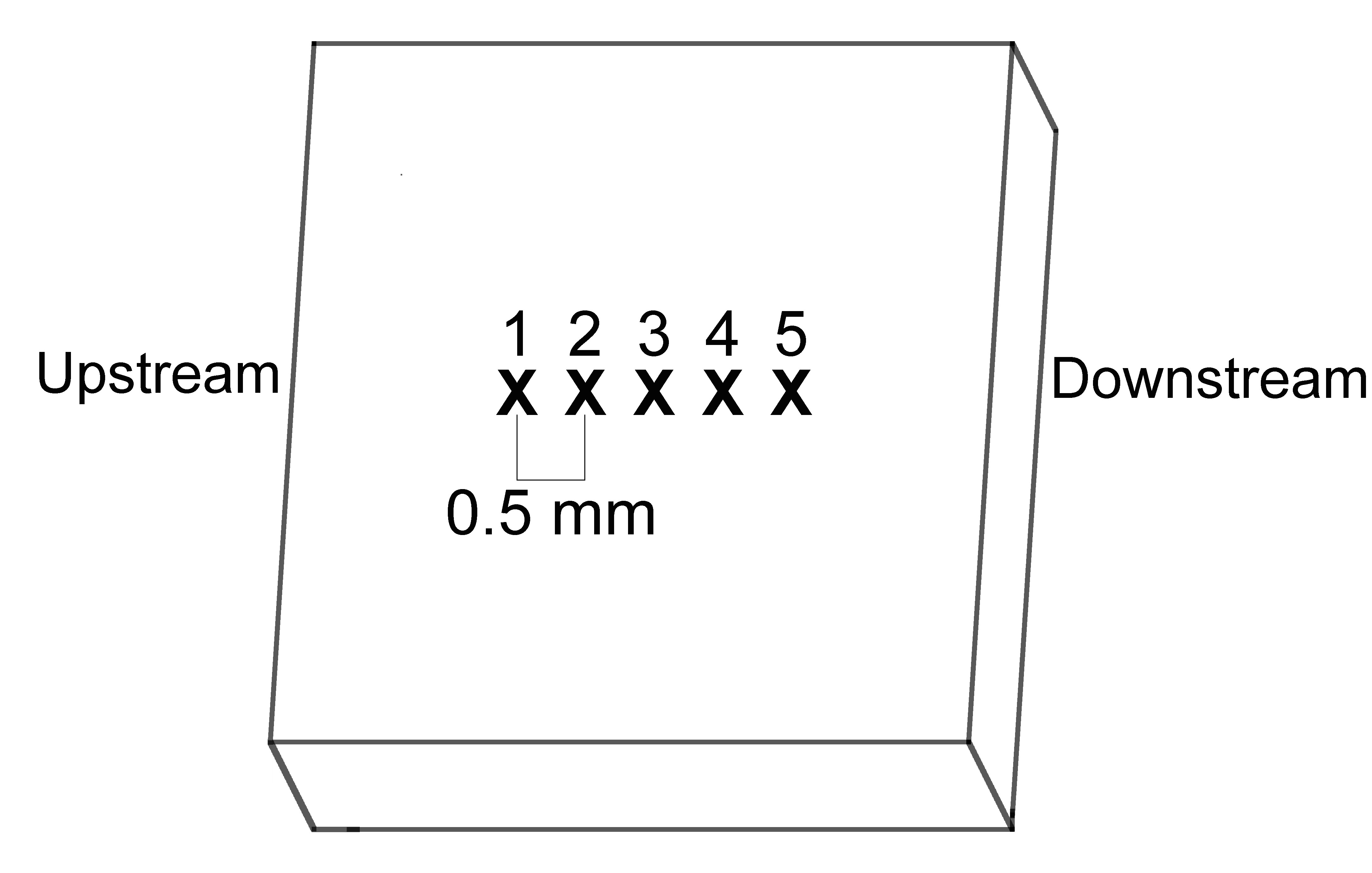

Supplement: Supplemental Material [file ZJOM_A_1949427_SM8111.zip › Supplementary/FIGS2 new version.jpg]

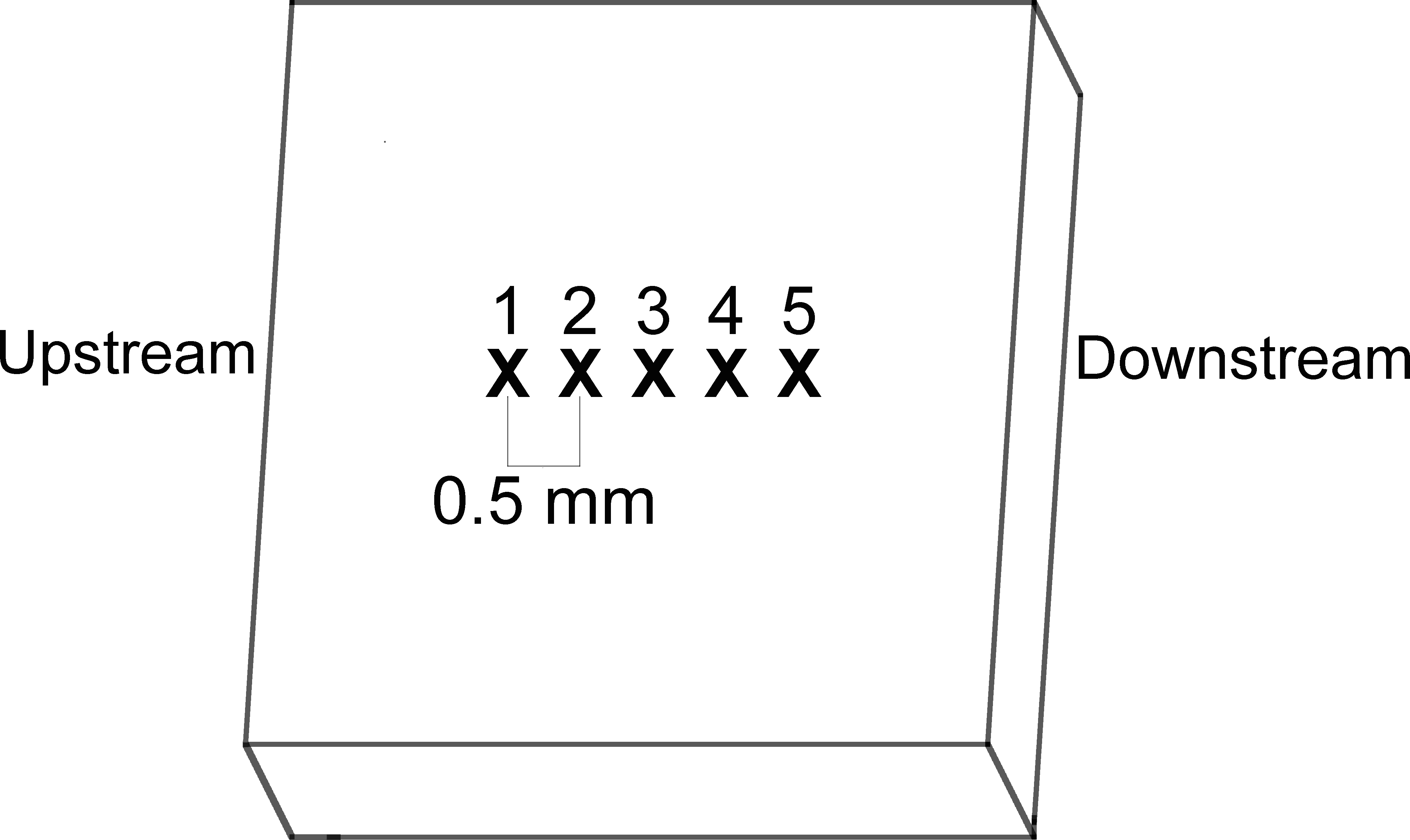

Supplement: Supplemental Material [file ZJOM_A_1949427_SM8111.zip › Supplementary/FIGS2.tif]

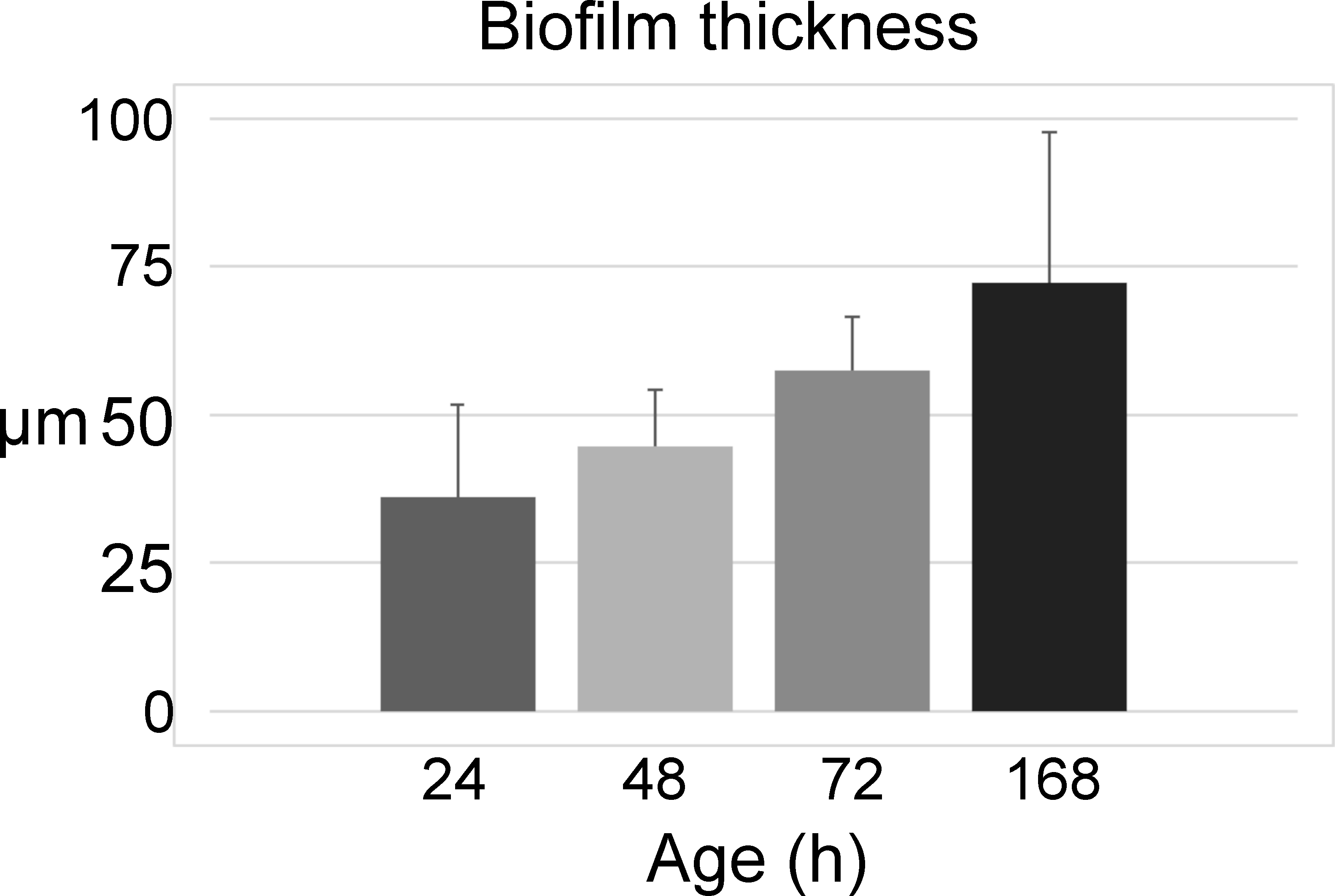

Supplement: Supplemental Material [file ZJOM_A_1949427_SM8111.zip › Supplementary/FIGS3.tif]

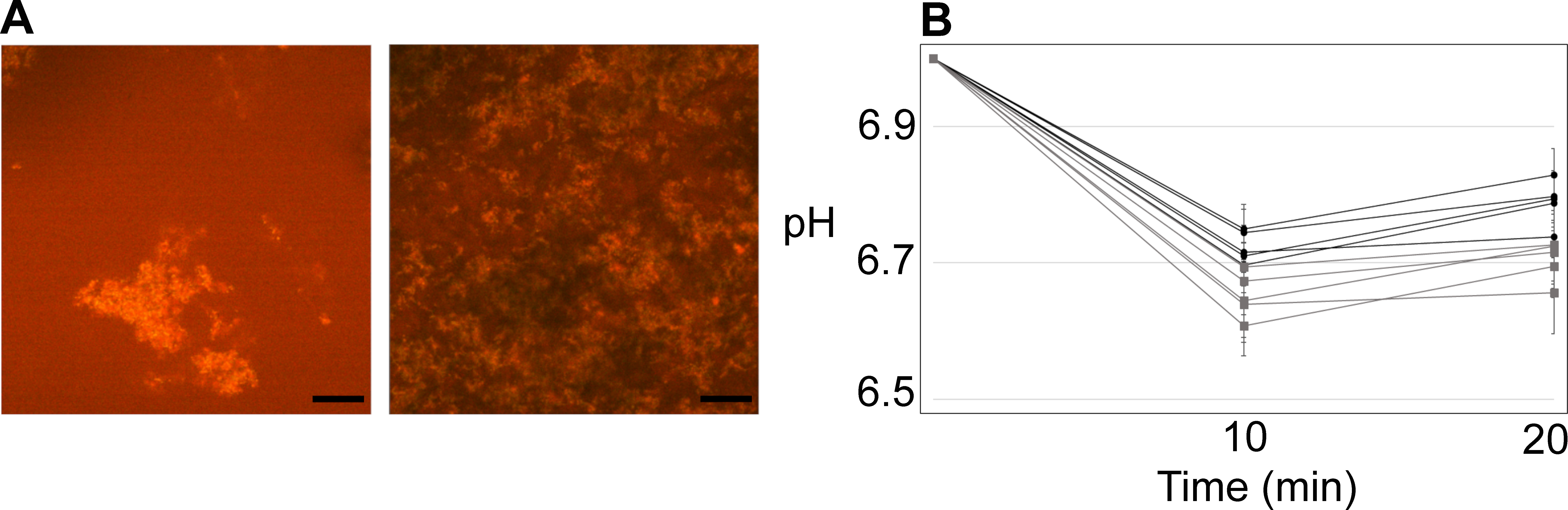

Supplement: Supplemental Material [file ZJOM_A_1949427_SM8111.zip › Supplementary/FIGS4.tif]

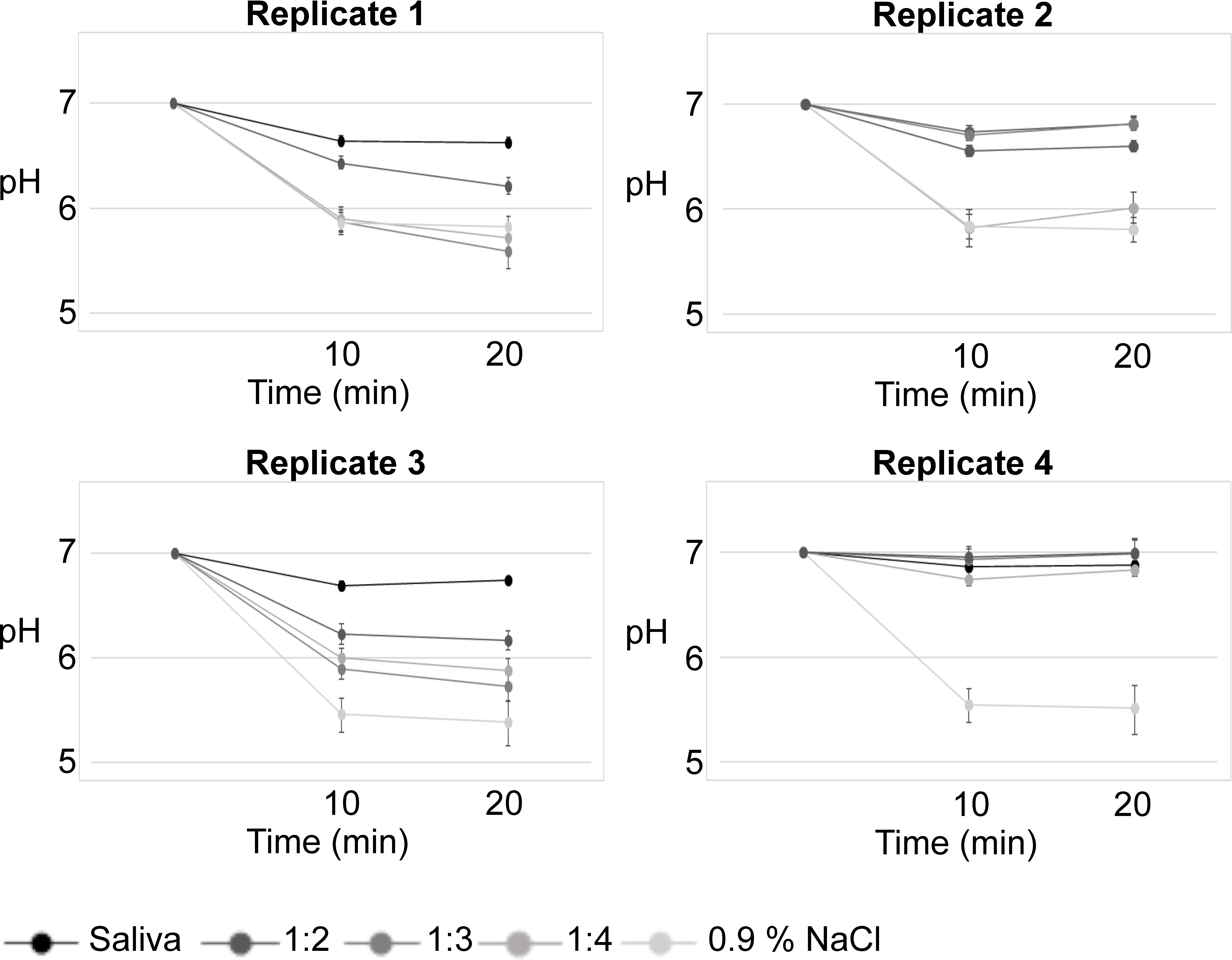

Supplement: Supplemental Material [file ZJOM_A_1949427_SM8111.zip › Supplementary/FIGS5.tif]

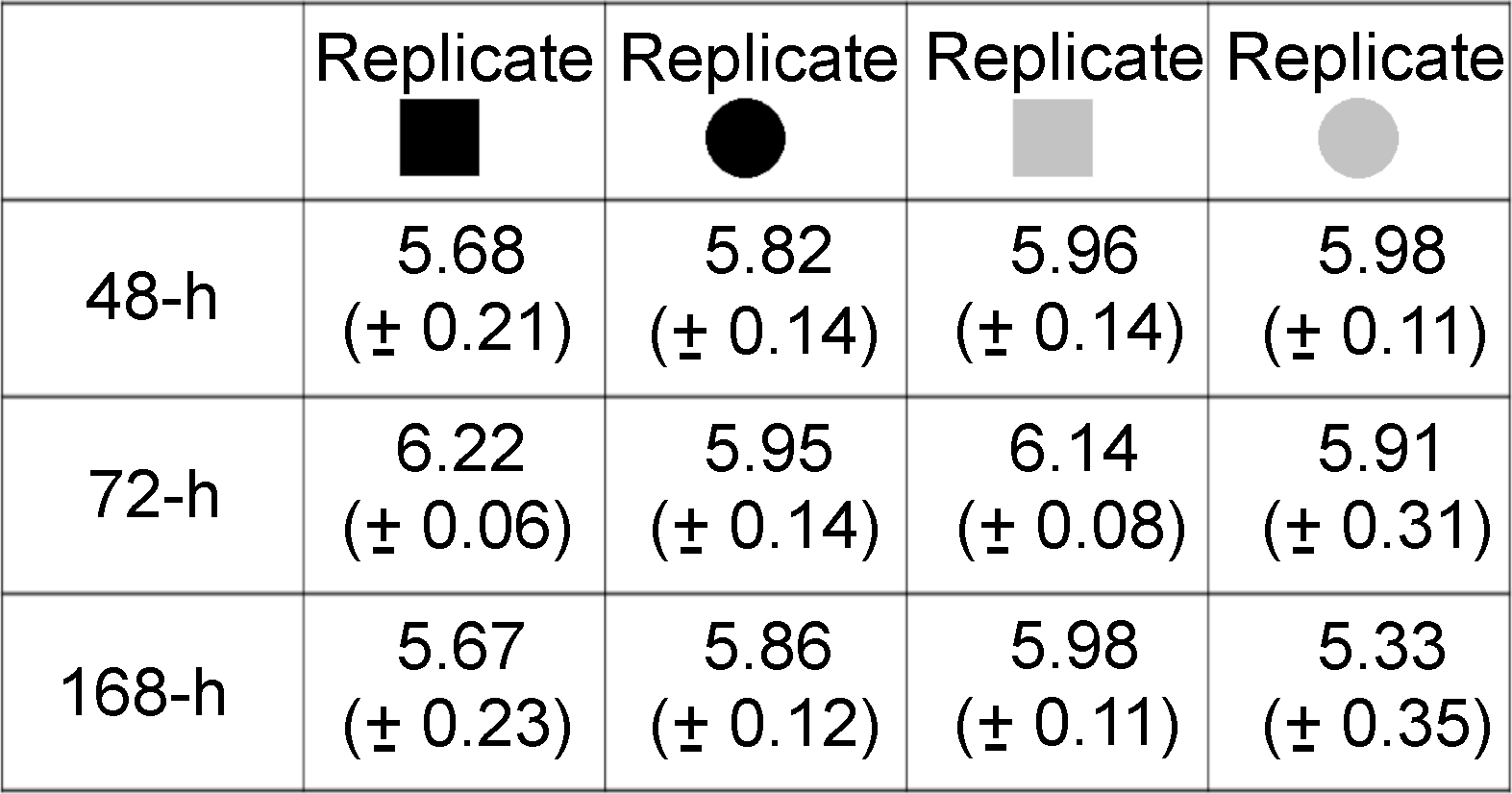

Supplement: Supplemental Material [file ZJOM_A_1949427_SM8111.zip › Supplementary/FIGS6.tif]

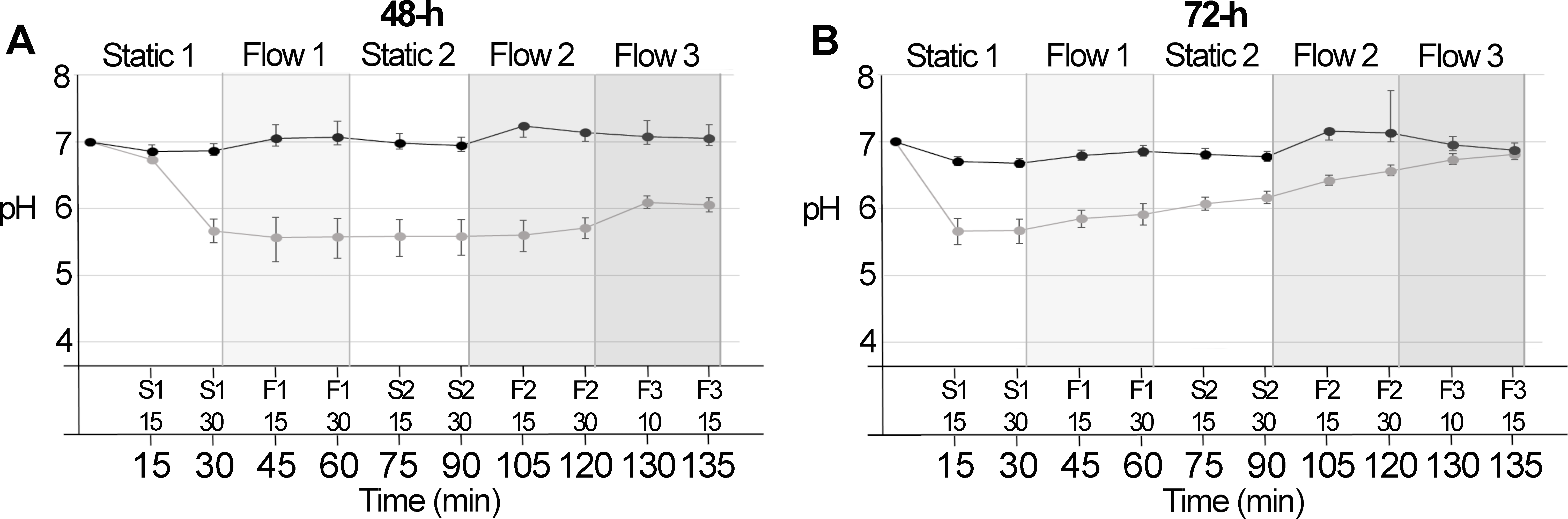

Supplement: Supplemental Material [file ZJOM_A_1949427_SM8111.zip › Supplementary/FIGS7.tif]

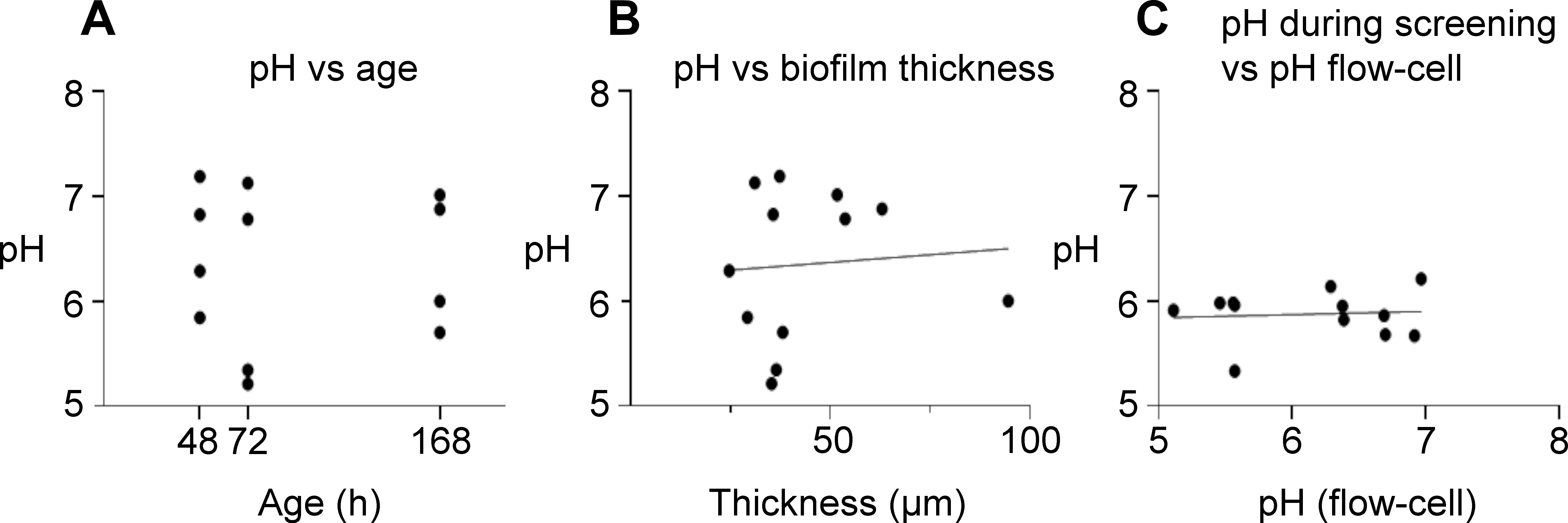

Supplement: Supplemental Material [file ZJOM_A_1949427_SM8111.zip › Supplementary/FIGS8 new version.jpg]
